# Perioperative Outcomes and Trends of Surgical Correction of Male Urethral Strictures: Results from the GRAND Study

**DOI:** 10.3390/jcm14072171

**Published:** 2025-03-22

**Authors:** Robert Bischoff, Julian Marcon, Gerald Bastian Schulz, Christian G. Stief, Patrick Keller, Lennert Eismann, Philipp Weinhold, Nikolaos Pyrgidis

**Affiliations:** Department of Urology, University Hospital of Munich (LMU), Marchioninistraße 15, 81377 Munich, Germany; robert.bischoff@med.uni-muenchen.de (R.B.); julian.marcon@med.uni-muenchen.de (J.M.); gerald.schulz@med.uni-muenchen.de (G.B.S.); christian.stief@med.uni-muenchen.de (C.G.S.); patrick.keller@med.uni-muenchen.de (P.K.); lennert.eismann@med.uni-muenchen.de (L.E.); philipp.weinhold@med.uni-muenchen.de (P.W.)

**Keywords:** urethral strictures, internal urethrotomy, urethroplasty, perioperative outcomes, surgical trends

## Abstract

**Background**: While various techniques for the surgical correction of urethral strictures exist, data on their trends and perioperative outcomes are limited. **Methods**: Data from the German Nationwide Inpatient Data (GRAND) registry (2005–2023) were used to estimate the trends, baseline characteristics, and perioperative outcomes of the surgical techniques for urethral stricture correction. **Results**: A total of 500,937 patients underwent surgery. Internal urethrotomy was the most frequently performed procedure (n = 413,095, 82%), followed by urethral dilatation (n = 39,619, 8%), meatoplasty (n = 30,774, 6%), urethroplasty with buccal mucosa (n = 12,351, 2%), urethral excision with primary anastomosis (n = 3428, 0.7%), urethroplasty with preputial skin (n = 1585, 0.3%), and drug-coated balloon dilatation (n = 85, <0.1%). In recent years, internal urethrotomy declined; urethroplasty was relatively stable, and drug-coated balloon dilatation emerged as a promising treatment modality. Internal urethrotomy and urethral dilatation were predominantly performed in older patients (median age of 71 years), while urethroplasty was performed in younger (56 years). Preputial skin urethroplasty had a shorter hospital stay compared to buccal mucosa (−0.4 days, *p* = 0.02), with no significant differences in transfusion or sepsis rates. **Conclusions**: Internal urethrotomy remains the most frequently used technique despite declining utilization. Preputial skin urethroplasty presents similar perioperative outcomes compared to buccal mucosa urethroplasty.

## 1. Introduction

Urethral strictures are a common urological condition characterized by the narrowing of the urethral lumen due to fibrosis and scar tissue formation, leading to variable degrees of urinary obstruction [1]. They can result from multiple etiologies, including iatrogenic injury (e.g., catheterization and endoscopic procedures), trauma, lichen sclerosus, infections, sexually transmitted diseases, and idiopathic causes [2]. The condition predominantly affects older males, with a prevalence of approximately 500 per 100,000 men over the age of 55 years [3]. Urethral strictures occur most frequently in the anterior part and mainly in the bulbar urethra [4]. Urethral strictures can significantly impact patients’ quality of life by causing obstructive urinary tract symptoms, recurrent infections, and, in severe cases, urinary retention [5]. The severity of symptoms often correlates with the degree of luminal narrowing, with most patients becoming symptomatic when the urethral caliber is reduced to less than 10 French [6]. Strictures wider than 10 French are often asymptomatic and discovered incidentally during endourological procedures [7].

The early and appropriate management of urethral strictures is essential to prevent complications and optimize long-term functional outcomes [8]. The available guideline recommendations suggest that patients with symptomatic strictures or patients with strictures less than 10 French should undergo surgical correction [9]. Nevertheless, the choice of the intervention is influenced by multiple factors including stricture length, location, etiology, and previous treatment history [10].

Direct vision internal urethrotomy is the first-line treatment due to its simplicity and accessibility [11]. Visually controlled dilatation of the urethra in an ambulatory setting can also be discussed with the patients, offering a pragmatic, immediately available solution [12]. Drug (paclitaxel)-coated balloon dilatation after standard dilatation has emerged as a viable alternative that is performed in Germany in a hospitalized setting for well-selected patients with recurrent short bulbar strictures [13]. However, the recurrence rates associated with all minimally invasive techniques lead to the application of more complicated treatment modalities, which offer more durable outcomes, such as free graft urethroplasty with buccal mucosa or preputial skin [14]. Alternatively, urethral excision with primary anastomosis can be discussed for selected short bulbar strictures [15].

Even though multiple techniques for the surgical management of urethral strictures exist, there are limited comprehensive data assessing their trends, perioperative outcomes, and evolving practices [16]. In this context, we aimed to perform an analysis of the perioperative outcomes and trends in the surgical correction of urethral strictures over the last two decades, aiming to provide valuable insights into the evolving landscape of stricture management and inform clinical decision-making to optimize patient care.

## 2. Methods

### 2.1. Data Source: German Nationwide Inpatient Data (GRAND)

This study utilized data from the German Nationwide Inpatient Data registry, maintained by the Federal Bureau of Statistics in Wiesbaden, Germany. The registry encompasses data on all hospitalizations in Germany between 2005 and 2023, excluding military, psychiatric, and forensic cases. Access to the data was obtained with the required approvals (LMU-4710-2022). The dataset comprises anonymized data stored at the Research Data Center of the Federal Bureau of Statistics. Our research team analyzed only aggregated summary results provided by the Research Data Center and did not have access to individual patient-level data. Ethical approval and patient consent were not required under German regulations. Our findings were reported based on the STROBE statement for cohort studies.

Since the implementation of the diagnosis-related group (DRG) system in 2005, German hospitals are mandated to submit inpatient data, including diagnoses, surgical procedures, and perioperative outcomes, to the institute for the hospital remuneration system. Diagnoses and perioperative outcomes are coded using the 10th revision of the International Classification of Diseases, German Modification (ICD-10-GM), while surgical procedures are coded using the German procedure classification (OPS). The standardization of documentation is ensured by the German Institute for Medical Documentation and Information.

### 2.2. Study Population and Outcomes

The study included all hospitalized male patients diagnosed with urethral stricture (ICD-10-GM: N35 or N99.1) who underwent free graft urethroplasty with buccal mucosa (OPS code: 5-584.72) or preputial skin (OPS code: 5-584.71), urethral excision with primary anastomosis (OPS code: 5-584.6), meatoplasty (OPS code: 5-581), urethral dilatation (OPS code: 8-139.0), direct vision internal urethrotomy (OPS code: 5-585), or drug-coated balloon dilatation (OPS code: 8-139.1) between 2005 and 2023. Patients undergoing staged correction of urethral strictures, as well as patients managed conservatively or those treated in an ambulatory setting, were excluded, since we did not have access to these data. Additional ICD-10-GM and OPS codes were used to identify comorbidities and inpatient complications. The primary aim of this study was to assess the trends and perioperative outcomes of patients undergoing surgical correction of urethral strictures. Secondary outcomes included comparing the perioperative outcomes of free graft urethroplasty with buccal mucosa versus preputial skin in terms of length of hospital stay, sepsis, and transfusion rates.

### 2.3. Statistical Analysis

We used multivariable logistic and linear regression models to assess the perioperative outcomes of free graft urethroplasty with buccal mucosa versus preputial skin in terms of length of hospital stay, sepsis, and transfusion rates. All models were adjusted for age, diabetes, chronic renal failure, hypertension, and obesity. Categorical data were presented as frequencies with proportions, and continuous data were presented as medians with interquartile ranges (IQRs). Odds ratios (ORs) with 95% confidence intervals (CIs) were calculated, and *p*-values below 0.05 were deemed statistically significant. The statistical analyses were performed by the Research Data Center based on R scripts developed by our team (source: Research Data Center, DRG Statistics 2005–2023). When less than three cases of an outcome were reported, these data were censored by the German Federal Statistical Office to prevent individual patient identification.

## 3. Results

### 3.1. Baseline Characteristics

The baseline characteristics of patients undergoing different surgical techniques for correction of urethral strictures are presented in Table 1. A total of 500,937 patients underwent surgery. Internal urethrotomy was the most frequently performed procedure (n = 413,095, 82%), followed by urethral dilatation (n = 39,619, 8%), meatoplasty (n = 30,774, 6%), urethroplasty with buccal mucosa (n = 12,351, 2%), urethral excision with primary anastomosis (n = 3428, 0.7%), urethroplasty with preputial skin (n = 1585, 0.3%), and drug-coated balloon dilatation (n = 85, <0.1%).

The median patient age varied across modalities and was higher in patients undergoing internal urethrotomy (71 years, with an IQR of 62–78) and urethral dilatation (71 years, with an IQR of 61–79) and lower in patients undergoing urethral excision with primary anastomosis (53 years, with an IQR of 35–68), urethroplasty with buccal mucosa (55 years, with an IQR of 40–68), and urethroplasty with preputial skin (56 years, with an IQR of 40–69). Concerning comorbidities, the most common were hypertension (n = 199,048, 48%) and benign prostatic hyperplasia (n = 130,556, 32%), followed by diabetes (n = 73,292, 18%). Median hospital stay ranged from 3 days for drug-coated balloon dilatation to 7 days for urethroplasty.

**Figure 1:**
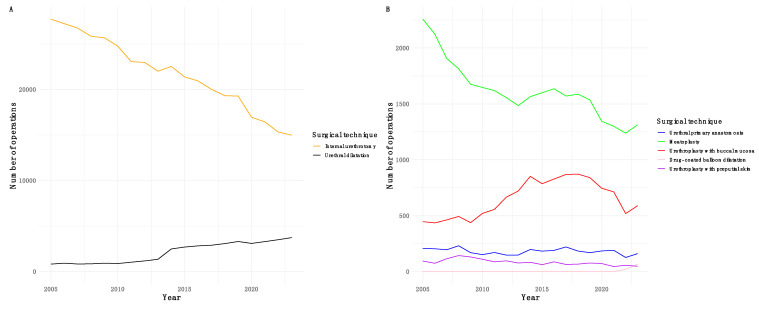
The annual trends of major techniques for the surgical correction of urethral strictures. Note that the *Y*-axis scale differs in (**A**,**B**).

Figure 1 illustrates the annual trends of surgical correction techniques for urethral strictures. Surgical correction of urethral strictures in a hospitalized setting has steadily declined in recent years. Internal urethrotomy remains the preferred treatment modality. Conversely, the frequency of advanced techniques like urethroplasty has remained relatively stable. Moreover, the use of drug-coated balloon dilatation has increased during the last few years.

### 3.2. Comparison of Outcomes: Urethroplasty with Buccal Mucosa Versus Preputial Skin

The perioperative outcomes of buccal mucosa and preputial skin urethroplasty are detailed in Table 2. Transfusion rates were slightly higher in patients undergoing urethroplasty with preputial skin (0.8%) compared to buccal mucosa urethroplasty (0.4%), but the difference did not reach statistical significance (OR: 1.81, 95% CI: 0.92 to 3.29, and *p* = 0.07). Similarly, the incidence of sepsis was low and comparable between the two groups (0.3% vs. 0.2%, OR: 1.2, 95% CI: 0.34 to 3, and *p* = 0.8). On the contrary, the length of hospital stay was significantly shorter for preputial skin urethroplasty compared to buccal mucosa urethroplasty (day difference: 0.4 days, 95% CI: 0.05 to 0.7, and *p* = 0.02).

## 4. Discussion

The present nationwide analysis provides a comprehensive evaluation of the baseline characteristics and trends in surgical correction techniques for urethral strictures. Our findings highlight that the number of cases that are annually performed is steadily decreasing. Still, internal urethrotomy remains the most common procedure, particularly in older patients, despite its declining use in recent years. Advanced techniques like urethroplasty, though performed less frequently, demonstrated stable utilization and low overall complication rates. Notably, urethroplasty with preputial skin was associated with a slightly shorter hospital stay compared to buccal mucosa urethroplasty. Still, no significant differences were observed in the rates of transfusion or sepsis between these techniques, indicating comparable safety profiles. Importantly, the observed increase in the use of drug-coated balloon dilatation during recent years suggests its emerging role as a minimally invasive option for select cases.

The trends in surgical correction of urethral strictures suggest a steady decline in the number of annual internal urethrotomies and meatoplasties performed in a hospitalized setting. Given that the worldwide incidence of urethral strictures is increasing and considering that the annual cases of urethroplasties have remained relatively stable, this trend might be explained by the fact that internal urethrotomies and meatoplasties are usually performed in an ambulatory setting [17,18]. Moreover, urethral dilatations remain a commonly performed procedure in the ambulatory setting for the management of urethral strictures [19]. In the present analysis, we included all urethral dilatations that were performed in a hospitalized setting. These were adjunct procedures to other endourological procedures such as transurethral resection of prostate or bladder tumors. [20]. Similarly, some of the internal urethrotomy cases might have been adjunct procedures to other surgeries. Still, internal urethrotomy continues to dominate as the most commonly utilized treatment modality, likely due to its simplicity and widespread adoption [21]. Importantly, the observed stable number of urethroplasty procedures suggests that the number of patients with complex or recurrent urethral strictures does not increase in Germany. On the contrary, in other European countries such as the UK, the annual urethrotomies is increasing [22]. Moreover, drug-coated balloon dilatations are gaining in popularity. Still, further long-term outcomes are expected with great interest to prove whether they can endure over time as an effective treatment modality [23].

Our findings are in line with previous studies, suggesting that buccal mucosa is the most common graft for urethroplasty [24]. Accumulating evidence also suggests that the perioperative outcomes of urethroplasty are low [25]. In our analysis, preputial skin urethroplasty was associated with a slightly shorter length of hospital stay, which is probably due to the pain and complications related to the buccal mucosa [26]. Nevertheless, it seems that the long-term safety and efficacy of both grafts are comparable. In particular, in a randomized controlled trial comparing buccal mucosa with penile skin as a graft for anterior strictures, no significant differences were observed between the two grafts [27]. On the contrary, a recent systematic review and meta-analysis suggested that buccal urethroplasty may provide better outcomes compared to penile skin graft urethroplasty [28]. Similarly, in patients with lichen sclerosus-related strictures, the use of penile skin graft is associated with poorer patency rates [29]. Still, it should be highlighted that the characteristics of each stricture display high variation, and a plethora of confounding factors may affect the outcomes [30]. Therefore, clear conclusions about the long-term efficacy of urethroplasty cannot be made. Based on the previous notion, our analysis focused on perioperative outcomes, underscoring the safety of both techniques.

It should be highlighted that it was beyond the scope of the present study to compare the perioperative outcomes of all surgical techniques for urethral strictures due to their specific indications and the complexity of each stricture. These variations underscore the need for individualized treatment approaches based on the nature of the stricture, patient characteristics, and surgeon expertise [31]. Available evidence suggests that internal urethrotomy, despite its high recurrence rates, remains the procedure of choice due to its simplicity and shorter recovery time [32]. Similarly, all endourological procedures may play an important role in specific clinical scenarios, particularly for patients unfit for urethroplasty [33]. Moreover, meatoplasty and urethral primary anastomosis serve as niche procedures for specific indications, such as distal strictures or traumatic injuries, respectively [34,35].

It should be stressed that randomized controlled studies or systematic reviews and meta-analyses comparing the different surgical procedures for male urethral strictures are lacking. Therefore, future research in urethral stricture management should focus on high-quality studies. Moreover, emerging minimally invasive techniques such as regenerative medicine approaches, including stem cell therapy and tissue-engineered grafts, have shown promising preliminary results and could revolutionize stricture treatment in the coming years [36]. It seems that the treatment trends of urethral strictures display significant variability worldwide [21]. In particular, some countries opt for conservative management approaches, such as intermittent self-catheterization, while others increasingly adopt sophisticated surgical interventions [37]. In North America, for instance, there is a trend toward the decreased use of repeat endoscopic procedures and the increased use of urethroplasty [18]. The latter underscores the importance of continued research into the optimal integration of existing and novel therapies, as well as the need for cost-effectiveness analyses to guide policy decisions and resource allocation in different healthcare settings [38].

This study represents, to the best of our knowledge, the largest analysis of baseline characteristics, perioperative outcomes, and trends in surgical correction techniques for urethral strictures. However, several limitations must be acknowledged. First, the retrospective design and reliance on administrative billing data may introduce inaccuracies due to coding errors or misclassifications. Additionally, critical clinical details such as the etiology, the location, the complexity or the length of the stricture, the applied surgical techniques (e.g., onlay or inlay urethroplasty and laser versus cold knife urethrotomy), the number of recurrences, and the operative time were not available for analysis. Given that our follow-up is restricted to the time in hospital, the absence of long-term data on functional outcomes, recurrence rates, and patient-reported quality of life limits the ability to draw comprehensive conclusions. While a comparison of outcomes between different urethroplasty techniques was performed, the lack of data on specific patient characteristics and intraoperative details may impact the interpretation of results. Importantly, we only had access to hospitalized cases; thus, the outcomes of ambulatory urethral dilatations could not be evaluated. Based on the previous notion, the real-life trends of some surgical techniques such as internal urethrotomy or drug-coated balloon dilatation might be even higher. The latter might also be supported by the fact that drug-coated balloon dilatation is not reimbursed by the German healthcare insurance in some cases. Based on the previous notion, as this study is specific to the German healthcare system, caution is advised when extrapolating these findings to other healthcare settings with varying surgical practices and resource availability.

## 5. Conclusions

The findings of this study provide valuable insights into the perioperative outcomes and trends in the surgical management of urethral strictures. Internal urethrotomy remains the most commonly performed procedure, though its use has declined in recent years. Advanced techniques, such as urethroplasty with buccal mucosa or preputial skin, have maintained stable utilization, highlighting their role in managing complex or recurrent strictures. Nevertheless, the choice of graft material should be guided by patient-specific factors and stricture complexity. Understanding the evolving trends and outcomes of urethral stricture surgeries is crucial for optimizing clinical decision-making and improving patient care. Overall, it should be highlighted that each surgical approach is associated with specific indications and limitations.

## Figures and Tables

**Table 1:** Baseline characteristics of the included patients. Variables are presented as the median (interquartile range) or frequencies with proportions. Censorship due to data protection are marked as XXX.

Characteristic	Urethral Primary Anastomosis, n = 3428	Urethral Dilatation, n = 39,619	Meatoplasty, n = 30,774	Urethroplasty with Buccal Mucosa, n = 12,351	Drug-coated Balloon Dilatation, n = 85	Urethroplasty with Preputial Skin, n = 1585	Internal Urethrotomy, n = 413,095
Age (years)	53 (35–68)	71 (61–79)	65 (44–74)	55 (40–68)	71 (58–77)	56 (40–69)	71 (62–78)
Benign prostatic hyperplasia	160 (4.7%)	11,299 (29%)	8849 (29%)	451 (3.7%)	XXX	XXX	130,556 (32%)
Diabetes	312 (9.1%)	8044 (20%)	4582 (15%)	1316 (11%)	11 (13%)	175 (11%)	73,292 (18%)
Chronic heart failure	66 (1.9%)	2898 (7.3%)	1048 (3.4%)	199 (1.6%)	6 (7.1%)	25 (1.6%)	21,236 (5.1%)
Chronic kidney disease	162 (4.7%)	5235 (13%)	1947 (6.3%)	481 (3.9%)	10 (12%)	61 (3.8%)	38,767 (9.4%)
Hypertension	1013 (30%)	20,517 (52%)	11,343 (37%)	4225 (34%)	33 (39%)	522 (33%)	199,048 (48%)
Obesity	181 (5.3%)	2554 (6.4%)	1623 (5.3%)	666 (5.4%)	3 (3.5%)	83 (5.2%)	22,861 (5.5%)
Hospital stay (days)	7 (5–11)	4.0 (2–7)	4 (2–6)	7 (6–11)	3 (2–4)	7 (6–10)	4 (3–7)
Reimbursement (EUR)	4789 (4583–5331)	2245 (1821–3250)	6856 (5884–7336)	2001 (1920–2191)	4711 (4363–5316)	2239 (1904–3045)	4789 (4583–5331)
Age group							
<50	1491 (43%)	4636 (12%)	8570 (28%)	4710 (38%)	12 (14%)	601 (38%)	39,245 (9.5%)
50–59	552 (16%)	3943 (10%)	3336 (11%)	2278 (18%)	11 (13%)	256 (16%)	40,169 (9.7%)
60–69	587 (17%)	7875 (20%)	6328 (21%)	2448 (20%)	16 (19%)	294 (19%)	89,700 (22%)
70–79	604 (18%)	12,597 (32%)	8197 (27%)	2409 (20%)	26 (31%)	329 (21%)	148,164 (36%)
Year of surgery							
2005	208 (6.1%)	821 (2.1%)	2258 (7.3%)	447 (3.6%)	0 (0%)	94 (5.9%)	27,718 (6.7%)
2006	204 (6.0%)	921 (2.3%)	2124 (6.9%)	435 (3.5%)	0 (0%)	74 (4.7%)	27,229 (6.6%)
2007	195 (5.7%)	829 (2.1%)	1906 (6.2%)	462 (3.7%)	0 (0%)	115 (7.3%)	26,736 (6.5%)
2008	231 (6.7%)	849 (2.1%)	1814 (5.9%)	493 (4.0%)	0 (0%)	143 (9.0%)	25,843 (6.3%)
2009	169 (4.9%)	918 (2.3%)	1676 (5.4%)	438 (3.5%)	0 (0%)	131 (8.3%)	25,676 (6.2%)
2010	151 (4.4%)	878 (2.2%)	1647 (5.4%)	521 (4.2%)	0 (0%)	110 (6.9%)	24,759 (6.0%)
2011	171 (5.0%)	1030 (2.6%)	1619 (5.3%)	556 (4.5%)	0 (0%)	87 (5.5%)	23,051 (5.6%)
2012	147 (4.3%)	1167 (2.9%)	1556 (5.1%)	666 (5.4%)	0 (0%)	96 (6.1%)	22,971 (5.6%)
2013	148 (4.3%)	1347 (3.4%)	1485 (4.8%)	720 (5.8%)	0 (0%)	77 (4.9%)	22,006 (5.3%)
2014	198 (5.8%)	2480 (6.3%)	1564 (5.1%)	852 (6.9%)	0 (0%)	83 (5.2%)	22,519 (5.5%)
2015	183 (5.3%)	2692 (6.8%)	1600 (5.2%)	786 (6.4%)	0 (0%)	62 (3.9%)	21,377 (5.2%)
2016	189 (5.5%)	2821 (7.1%)	1636 (5.3%)	828 (6.7%)	0 (0%)	87 (5.5%)	20,935 (5.1%)
2017	220 (6.4%)	2888 (7.3%)	1570 (5.1%)	869 (7.0%)	0 (0%)	63 (4.0%)	20,009 (4.8%)
2018	184 (5.4%)	3076 (7.8%)	1587 (5.2%)	872 (7.1%)	0 (0%)	67 (4.2%)	19,317 (4.7%)
2019	169 (4.9%)	3297 (8.3%)	1536 (5.0%)	840 (6.8%)	0 (0%)	76 (4.8%)	19,275 (4.7%)
2020	185 (5.4%)	3089 (7.8%)	1344 (4.4%)	745 (6.0%)	0 (0%)	72 (4.5%)	16,947 (4.1%)
2021	190 (5.5%)	3295 (8.3%)	1300 (4.2%)	713 (5.8%)	0 (0%)	45 (2.8%)	16,434 (4.0%)
2022	126 (3.7%)	3495 (8.8%)	1238 (4.0%)	519 (4.2%)	20 (24%)	56 (3.5%)	15,318 (3.7%)
2023	160 (4.7%)	3723 (9.4%)	1314 (4.3%)	589 (4.8%)	65 (76%)	47 (3.0%)	14,975 (3.6%)

**Table 2:** Multivariable linear and logistic regression analysis comparing free graft urethroplasty with buccal mucosa versus preputial skin. All models are adjusted for age, diabetes, chronic renal failure, hypertension, and obesity. The bold cells indicate statistically significant *p*-values. CI: confidence interval.

Outcome	Urethroplasty with Buccal Mucosa		Urethroplasty with Preputial Skin	
	Cases	Estimate (95% CI), *p*-Value	Cases	Estimate (95% CI), *p*-Value
**Transfusion**	52 (0.4%)	—	12 (0.8%)	1.81 (0.92, 3.29), 0.07
**Sepsis**	27 (0.2%)	—	4 (0.3%)	1.2 (0.34, 3), 0.8
**Length of hospital stay**	7 (6–11)	—	7 (6–10)	**−0.4 (−0.7, −0.05), 0.02**

## Data Availability

The original contributions presented in this study are included in the article. Further inquiries can be directed to the corresponding author(s).
